# 5-ALA/SFC Mitigates Tau Toxicity via Lowering Oxidative Stress in a *Drosophila* Model of Tau Toxicity

**DOI:** 10.3390/life16050725

**Published:** 2026-04-24

**Authors:** Arisa Tamura, Marie Noguchi, Naoko Nozawa, Emiko Suzuki, Kanae Ando

**Affiliations:** 1 Department of Biological Sciences, Graduate School of Science, Tokyo Metropolitan University, Hachioji, Tokyo 192-0397, Japan; tamura-arisa@ed.tmu.ac.jp (A.T.); emikosuz@tmu.ac.jp (E.S.); 2 Division of Pharmaceutical Research, SBI Pharmaceuticals Co., Ltd., Tokyo 106-6020, Japan; nnozawa@sbigroup.co.jp; 3 Department of Biological Sciences, Faculty of Science, Tokyo Metropolitan University, Hachioji, Tokyo 192-0397, Japan

**Keywords:** mitochondria, 5-ALA/SFC, *Drosophila* model of tau toxicity, oxidative stress, neurodegeneration

## Abstract

Mitochondrial dysfunctions contribute to the pathogenesis of tauopathies, a group of neurodegenerative diseases with abnormal accumulation of microtubule-associated protein tau. The combination of 5-aminolevulinic acid (5-ALA) and sodium ferrous citrate (SFC) is known to improve mitochondrial functions. Here, we report that 5-ALA combined with SFC (5-ALA/SFC) improves mitochondrial functions and mitigates neurodegeneration in transgenic *Drosophila* expressing human tau. We found that tau reduces ATP levels, decreases mitochondrial distribution to neurites, and increases mitochondrial reactive oxygen species (ROS). Expression of oxidative phosphorylation (OXPHOS) genes was upregulated, and activities of complexes I and IV were elevated. Feeding 5-ALA/SFC to tau flies lowers oxidative damage without correcting OXPHOS activities or mitochondrial distribution. 5-ALA/SFC treatment suppressed pathological tau phosphorylation and mitigated tau-induced neurodegeneration. These results suggest that 5-ALA/SFC attenuates a neurodegenerative pathway involving tau, mitochondria, and ROS.

## 1. Introduction

Aging increases the risk of Alzheimer’s disease (AD) and other neurodegenerative diseases, and the number of people suffering from AD is rapidly increasing [1,2]. Many of these disorders are pathologically characterized by the abnormal accumulation of the microtubule-associated protein tau, collectively called tauopathy [3]. Tau is an intrinsically disordered protein; however, in tauopathy brains, tau is hyperphosphorylated and aggregated [4,5]. Accumulation of such abnormal tau causes neurodegeneration [6], while the mechanism by which tau causes neurodegeneration is not fully understood.

Mitochondria play critical roles in neuronal functions by providing most of the ATP required for brain neurons through the electron transfer chain and oxidative phosphorylation (OXPHOS) [7,8]. During OXPHOS, complexes I, III, and IV pump protons into the intermembrane space, generating the mitochondrial membrane potential that drives complex V, the ATP synthase, to produce ATP [9]. Electron leakage from the chain forms superoxide anions, which are subsequently converted into hydrogen peroxide (H_2_O_2_), hydroxyl radicals (•OH), and superoxide (O_2_^−^) [7]. These reactive oxygen species (ROS) are primarily produced at complexes I and III [7,10], which can cause oxidative damage. Mitochondria serve as a Ca^2+^ sink to maintain Ca^2+^ homeostasis for neuronal activity [11], regulate stress responses [12], and maintain proteostasis [13,14]. Mitochondrial abnormalities are observed in the brains of patients suffering from AD and other neurodegenerative diseases, and boosting mitochondrial functions may delay or treat neurodegenerative conditions [7].

5-aminolevulinic acid (5-ALA) has emerged as a potential strategy to improve mitochondrial functions [15]. 5-ALA is a precursor of heme and is synthesized from succinyl-CoA and glycine in mitochondria [16]. 5-ALA is crucial for mitochondrial functions, which rely on heme in the OXPHOS complexes II, III, IV, and cytochrome c [17]. A combination of 5-ALA and sodium ferrous citrate (SFC) (5-ALA/SFC) boosts mitochondrial functions in cultured fibroblasts, *Drosophila*, and mice [18,19,20,21,22]. A combination of 5-ALA and SFC, but not 5-ALA or SFC alone, increases expression of OXPHOS subunits, oxygen consumption rate, and ATP production in fibroblasts [18]. 5-ALA/SFC treatment protects against age-related functional declines and lethality caused by CI deficiency in *Drosophila* [22,23]. In a triple transgenic mouse model of AD (3x-Tg), 5-ALA/SFC feeding increased mitochondrial protein levels [24]. However, the effects of 5-ALA/SFC on tau toxicity remain unknown.

In this study, we investigated tau-induced changes in mitochondria and the effects of 5-ALA/SFC on them, as well as tau toxicity. A *Drosophila* model of tau toxicity has been established by expressing human tau in neurons or eyes [25], which recapitulate key pathological features of tauopathy such as tau phosphorylation at disease-related sites and neurodegeneration [25,26,27,28,29]. With this model, we found that feeding 5-ALA/SFC reduced ROS and mitigated tau-induced neurodegeneration.

## 2. Materials and Methods

### 2.1. Fly Stocks

Transgenic fly lines carrying human 0N4R tau were a kind gift from Dr. Mel Feany (Harvard Medical School) [25]. The GMR-Gal4 (RRID: BDSC_1104) and elav-Gal4 (RRID: BDSC_458) were obtained from the Bloomington Drosophila Stock Center. Flies with UAS-mito-roGFP-Grx1 (RRID: BDSC_67664) [30] were obtained from Bloomington Stock Center.

Flies were kept at 25 °C with a 12 h cycle of light and dark conditions. Adult flies were transferred to fresh vials after eclosion and transferred to fresh food vials every 2 to 3 days. Fly genotypes are listed in Table 1.

### 2.2. 5-ALA/SFC Feeding

Flies were raised on cornmeal food (10% glucose, 9% cornmeal, 4% yeast, 0.8% agar, 1% nipagin, 0.3% propionic acid [*w*/*v*]) without 5-ALA (hydrochloride)/SFC or with the indicated concentrations of 5-ALA (hydrochloride)/SFC. 5-ALA/SFC solution was prepared by combining 5-ALA dissolved in double-distilled water and SFC dissolved in 10% HCl at a 1:0.05 ratio, then diluted with double-distilled water to a concentration 10 times higher than the indicated final concentrations. Then, 1 volume of 5-ALA/SFC solution was combined with 9 volumes of cornmeal food, vortexed thoroughly, and left to solidify. Food added with the double-distilled water was used as a control.

### 2.3. ROS Analysis with Mito-roGFP2-Grx1

Flies were anesthetized with CO_2_, and brains were dissected in PBS-NEM (137 mM NaCl, 2.7 mM KCl, 10 mM Na_2_HPO_4_, 1.8 mM KH_2_PO_4_, 20 mM NEM, pH 7.4). The brains were incubated for 5 min in PBS-NEM at room temperature and then fixed for 8 min in 4% PFA/PBS-NEM. Brains were washed with PBS-NEM three times, imaged on the same day through a laser confocal microscope (Nikon C2, Nikon, Shinagawa, Tokyo, Japan), and analyzed with ImageJ ver. 2.16.0 [31]. Flies used for this assay were 3 days old after eclosion.

### 2.4. ATP Assay

ATP was extracted as previously described [32] using 6 M guanidine-HCl in the extraction buffer (100 mM Tris and 4 mM EDTA, pH 7.8) and the ATP Determination Kit (Cat#: A22066, Invitrogen, Carlsbad, CA, USA). Luminescence was measured on an EnSpire Multimode Plate Reader (PerkinElmer, Shelton, CT, USA). Relative ATP levels were determined by normalizing luminescence values to total protein concentrations, using the Bradford method. Flies used for the ATP assay were 10 days old (GMR-gal4:UAS-tau), 0 to 1 day old, and 46 to 49 days (elav-gal4:UAS-tau) after eclosion.

### 2.5. Mitochondrial DNA (mtDNA)/Nuclear DNA (nDNA) Ratio

mtDNA extraction for mitochondrial quantification was performed following a modified protocol [23]. Total DNA was purified from 60 fly heads using QIAamp DNA Mini Kit (Cat#: 51304, QIAGEN, Hilden, Germany). The expression levels of target genes were measured using THUNDERBIRD SYBR qPCR Mix (Cat#: QPS-201, TOYOBO, Osaka, Japan) on a Thermal Cycle Dice TP800 (Takara Bio, Kusatsu, Shiga, Japan). For mtDNA quantification, the average expression levels of *mt:COI*, *mt:COIII*, and *mt:CytB* were used. nDNA was quantified using the mean expression levels of *Sicily*, *Act5C*, and *rp49.* Analysis of the mtDNA/nDNA ratio was calculated by following the ΔΔCt method used for RT-qPCR analysis [33]. Flies used for mtDNA analysis were 1–2 days old after eclosion. Primer sequences (5′-3′) were as follows: *mt:COI* (F, AAAGTTGACGGTACACCTGG) (R, AGGAACACTTTCAATTACAATCGG), *mt:COIII* (F, CACGAGAAGGAACATACC) (R, GCGGGTGATAAACTTCTG), *mt:CytB* (F, GAAAATTCCGAGGGATTCAA) (R, AACTGGTCGAGCTCCAATTC), *Sicily* (F, AGTGGAGAAATACGACTACGAGA) (R, GATGGCCGCTAACCGATCTG), *Act5C* (F, AAGCTGTGCTATGTTGCCCT) (R, ATTCCCAAGAACGAGGGCTG), and *rp49* (F, ATCGTGAAGAAGCGCACCAA) (R, GTCGATACCCTTGGGCTTGC).

### 2.6. Blue Native PAGE (BN-PAGE), High-Resolution Clear Native PAGE (hrCNE), and In-Gel Activity Assay (IGA)

BN-PAGE to analyze the mitochondrial respiratory complex was carried out as described, with a modification [23,34,35]. In total, 120 fly heads were homogenized in 1 mL of chilled mitochondrial isolation medium (250 mM sucrose, 0.15 mM MgCl_2_, 10 mM Tris-HCl (pH 7.4)). After removing debris by centrifuging twice for 15 min at 600 *g* at 4 °C, mitochondria were collected by centrifuging for 5 min at 13,000 *g* at 4 °C. Protein levels were analyzed by bicinchoninic acid (BCA) assay (Cat#:126625-50ML, Sigma, Kawasaki, Kanagawa, Japan), and 17–96 µg per lane was used. Proteins were dissolved in sample buffer (50 mM NaCl, 20 mM Tris-HCl (pH 7.4), 1% Triton-X100) and centrifuged for 5 min at 14,000 rpm at 4 °C. The supernatants with loading dye (5% Coomassie Blue G, 1 M aminohexanoic acid, 100 mM Bis-Tris) were loaded onto 3–12% NativePAGE gels (NativePAGE Novex Bis-Tris Gel System Cat#:BN1001BOX, Life Technologies, Carlsbad, CA, USA).

For hrCNE, mitochondrial fractions were incubated in sample buffer (50 mM NaCl, 50 mM imidazole/HCl, 2 mM 6-aminohexanoic acid, 1 mM EDTA (pH 7.0), DDM (2.5 g/g protein), and digitonin (4 g/g protein)) for 10 min on ice. After centrifugation for 15 min at 10,000 *g* at 4 °C, the supernatants were mixed with 10× loading dye solution (50% glycerol, 0.1% Ponceau S) and separated on a 3–12% *NativePAGE* Novex Bis-Tris Gel System (Cat#:BN1001BOX, Life Technologies, Carlsbad, CA, USA). The gels were stained with a colloidal blue staining kit (Thermo Fisher Scientific, Waltham, MA, USA).

IGA was carried out as described previously, with a modification [36]. Mitochondrial fractions were separated by BN-PAGE. To detect CI activity, the gel was incubated with CI activity substrate (0.01% (*w*/*v*) NADH, 0.025% (*w*/*v*) Nitrotetrazolium Blue chloride (NTB), 2 mM Tris-HCl (pH 7.4)) for 15 min at room temperature and transferred to 10% acetic acid. To analyze CII activity, the gel was incubated in CII activity substrate (20 mM Sodium Succinate, 0.025% (*w*/*v*) NTB, 200 µM Phenazine Methosulphate, 5 mM Tris-HCl (pH 7.4)) for 40 min at 37 °C, then transferred to 10% acetic acid. For CIV activity, the gel was incubated with CIV activity substrate (0.05% (*w*/*v*) diaminobenzidine (DAB), 0.01% (*w*/*v*) cytochrome c, 45 mM phosphate buffer (pH 7.4)) overnight at room temperature. For CV activity, the gel was incubated with CV activity substrate (35 mM Tris, 270 mM glycine, 14 mM MgSO_4_, 10 mM ATP, 0.2% (*w*/*v*) Pb(NO_3_)_2_) overnight at room temperature, followed by incubation with 50% methanol. Flies used for IGA were 2–3 days old after eclosion.

### 2.7. Quantitative Reverse Transcription PCR (qRT-PCR)

In total, 30 flies were collected and frozen with liquid nitrogen, and heads were collected and homogenized. Total RNA was extracted in ISOGEN (Cat#:311-02501, Nippon Gene, Chiyoda, Tokyo, Japan). RNA concentration was measured with the NanoDrop One Spectrophotometer (Thermo Scientific, Waltham, MA, USA). 500 ng of total RNA was subjected to cDNA synthesis using ReverTra Ace qPCR RT Master Mix with gDNA Remover. Quantitative real-time PCR was performed using THUNDERBIRD SYBR qPCR Mix on a Thermal Cycler Dice TP800 (Takara Bio, Kusatsu, Shiga, Japan). The expression level of each gene was normalized either to the expression level of Actin or rp49. Flies used for RT-qPCR were 10 days old after eclosion. Primer sequences (5′-3′) were as follows: *Actin* (F, TGCACCGCAAGTGCTTCTAA) (R, TGCTGCACACTCCAAACTTCCA), *rp49* (F, ATCGTGAAGAAGCGCACCAA) (R, GTCGATACCCTTGGGCTTGC), *ND42* (F, ACACAGTCGCCCAATCTACTC) (R, GCTGTACTTCTTCGTCTTGTACG), *SdhA* (F, TGTACGACACGGTCAAGGG) (R, TTCTCCAGCTCAATGACAGCC), *RFeSP* (F, GTCCTCTCCACGGGATTGAA) (R, TGAAGGTGTTAACCAGGCCC), Cyt-c-p (F, AAGCACAAGGTTGGACCCAA) (R, AAGATCATCTTGGTGCCGGG), *COX4* (F, TACGATGAGCTGCCCGTTAC) (R, GGTTGATTTCCAGGTCGATGAT), and *blw* (F, CCGTTTCCGTGTGGGAATCAA) (R, AGAGCGGTCTTACCAGTCTGA).

### 2.8. Electron Microscopy

Flies were anesthetized with CO_2_, and their heads were cut into halves in primary fixative solution (2% PFA and 2.5% in 0.1 M Na cacodylate buffer). The heads were incubated in the fixative solution for 2 h at room temperature, followed by overnight incubation at 4 °C. The samples were washed with 3% sucrose in 0.1 M Na cacodylate buffer three times for 5 min each on ice. The samples were then incubated in ice-cold secondary fixative solution (1% OsO4 in 0.1 M Na cacodylate buffer) for 1 h on ice and washed with H_2_O three times for 5 min on ice. After washing, samples were dehydrated with ethanol and embedded in an Epon mixture (Cat#:3402, NEM, Tokyo, Japan). Ultra-thin sections of 50 nm were sliced sagittally and collected on copper grids. The sections were stained with 2% uranyl acetate in 70% ethanol and Reynolds’ lead citrate solution. Images were captured under a transmission electron microscope, JEM-1400Plus (JEOL Ltd., Akishima, Tokyo, Japan), and analyzed manually. Flies used for electron microscopic analysis were 48 to 50 days old after eclosion.

### 2.9. Anti-4-Hydroxynonenal (4-HNE) Staining

Brains were dissected in cold Schneider’s Drosophila Medium (Cat#:21720024, Thermo Fisher Scientific, Waltham, MA, USA). The brains were fixed in 4% PFA for 40 min at room temperature, then washed with 0.1% PBST (0.1% Triton in PBS) three times for 5 min. Then, the samples were blocked with 1% NGS in 0.1% PBST for 1 h at room temperature. After blocking, the samples were incubated with anti-4-HNE antibody (Cat#:46545, Abcam, Cambridge, UK) (1:100) in 1% normal goat serum (Cat#:551-76253, FUJIFILM, Tokyo, Japan) in 0.1% PBST at 4 °C overnight. The next day, the samples were washed with 0.1% PBST three times for 5 min, then incubated with Alexa-Fluor 647-conjugated anti-rabbit IgG (1:500) for 3 h at room temperature. The samples were again washed three times with 0.1% PBST for 5 min each and mounted. Images were captured using a Zeiss LSM 710 laser confocal microscope (Zeiss, Oberkochen, Germany) and analyzed with ImageJ ver. 2.16.0 [31]. Flies used for this assay were 48 to 50 days old after eclosion.

### 2.10. Histological Analysis

Neurodegeneration in the lamina was analyzed as previously described [37,38]. Fly heads were dissected and fixed in Bouin’s fixative solution for 48 h at room temperature. The heads were washed three times with 50 mM Tris/150 mM NaCl and incubated overnight at room temperature. Then, the samples were dehydrated with ethanol and subjected to paraffin embedding. Serial sections were sliced (7 μm) and stained with hematoxylin and eosin. Images were captured under a bright field of a Keyence microscope BZ-X700 (Keyence, Osaka, Japan), and the area of vacuoles in the lamina was analyzed with ImageJ ver. 2.16.0 [31] as previously described [38]. Flies were 10 days old after eclosion.

### 2.11. Western Blotting

Fifteen flies were collected and frozen with liquid nitrogen. Heads were homogenized in SDS-Tris-Glycine sample buffer, and the lysate was loaded into the lanes of a 10% Tris-Glycine gel. The gel was then transferred to a PVDF membrane (Cat#:IPVH00010, Merck Millipore, Burlington, MO, USA), and the membrane was blocked with 5% skim milk (Morinaga, Minato, Tokyo, Japan) for 1 h at room temperature. The membrane was incubated with antibodies in 5% skim milk and then visualized using Immobilon Western Chemiluminescent HRP Substrate (Cat#: WBKLS0050, Merck Millipore, Burlington, MO, USA). Images were captured using the Fusion FX (Vilber, Marne-la-Vallée, France), and band intensity was analyzed with ImageJ ver. 2.16.0. Flies used for Western blotting were 10 days old after eclosion. Anti-tau (T46, Cat#:13–6400, Thermo Fisher Scientific, Waltham, MA, USA), anti-pSer202, anti-pSer262 (pSer262, Cat#:ab92627, Abcam, Cambridge, UK), and anti-actin (Cat#:A2066, Sigma, Kawasaki, Kanagawa, Japan) were purchased. Anti-pSer202 (CP13) was a kind gift from Dr. Peter Davis (Albert Einstein College of Medicine).

### 2.12. Statistical Analysis

The number of replicates, n, precision measurements, and the meaning of error bars are indicated in the figure legends. For pairwise comparisons, Student’s *t*-test was performed with Microsoft Excel (Microsoft). For multiple comparisons, data were analyzed using one-way ANOVA with Dunnett’s post hoc test in the GraphPad Prism 6.0 software (GraphPad Software, Inc., La Jolla, CA, USA). Results with a *p*-value of less than 0.05 were considered to be statistically significant.

## 3. Results

### 3.1. Effects of Tau on Mitochondria

#### 3.1.1. Tau Increases Mitochondrial ROS in Neurons

**Figure 1:**
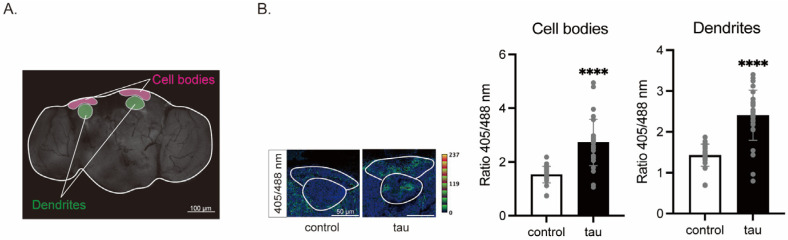
Tau increases mitochondrial ROS in neurons. Relative oxidation levels of mitochondrial glutathione redox potential biosensor mito-roGFP2-Grx1 expressed in neurons with or without co-expression of tau. Male flies at 3 days after eclosion were used. (**A**) Schematic representation of the cell body region and the dendritic region in the mushroom body of the fly brain. (**B**) Representative ratiometric images of mito-roGFP2-Grx1 (**left**), and quantitation of oxidized/reduced roGFP (**right**). Data are expressed as mean ± SE, *n* = 29; **** *p* < 0.0001; Student’s *t*-test.

We set out to characterize tau-induced changes in mitochondria in neurons. First, we analyzed ROS levels in tau-expressing neurons in the fly brain by using a mitochondrial redox sensor to detect the glutathione reducing pool, mito-roGFP2-Grx1 [30]. The redox-sensitive green fluorescent proteins (roGFPs) contain an oxidizable dithiol pair on their surface, and oxidation-induced disulfide bond formation converts mito-roGFP2-Grx1 from 488 nm to UV excitation [39]. mito-roGFP2-Grx1 [30] was co-expressed with human tau in neurons using the pan-neuronal driver, *elav-Gal4*, and signals in the mushroom body, where the cell body region and dendritic region are easily located, were quantified (Figure 1A). We found that tau expression causes higher oxidation, both in the dendrites and cell bodies of the mushroom body (Figure 1B), indicating that tau expression enhances mitochondrial ROS production.

#### 3.1.2. Tau Expression Reduces ATP Levels

**Figure 2:**
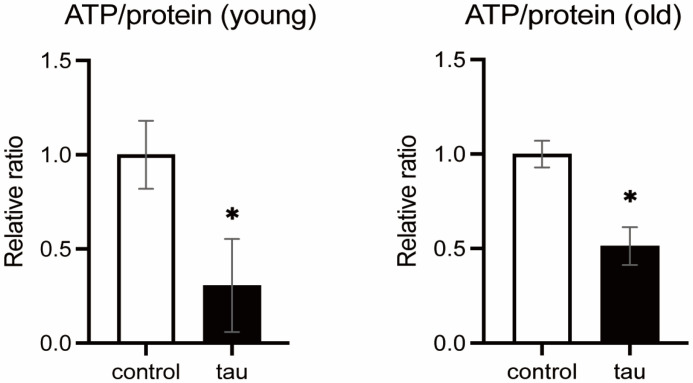
Tau expression reduces ATP levels. 10 heads of male flies expressing tau in neurons at 2 days after eclosion (**left**, young) and 46–49 days after eclosion (**right**, old) were homogenized and subjected to ATP assay. ATP levels were normalized by protein levels and expressed as a relative ratio. Data are expressed as mean ± SE, *n* = 3; * *p* < 0.05; Student’s *t*-test.

Next, we analyzed ATP levels in the heads of flies with or without tau expression in neurons at a young age (2 days after eclosion) and old age (46–49 days after eclosion). ATP levels were significantly reduced in tau-expressing flies compared to controls, in both young and aged cohorts (Figure 2).

#### 3.1.3. Tau Does Not Reduce the Number of Mitochondria

**Figure 3:**
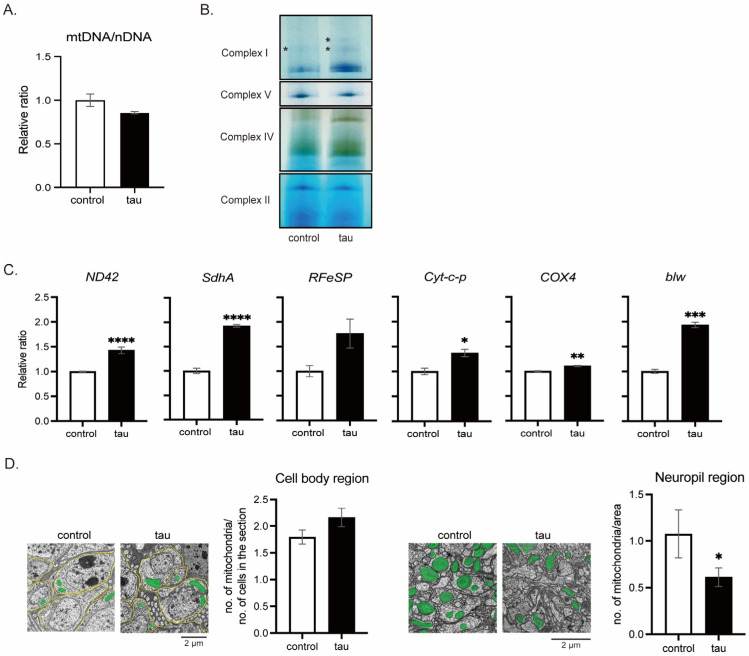
Tau expression upregulates the expression of the OXPHOS coding genes. (**A**) Levels of mtDNA and nDNA in heads of male flies with neuronal tau expression were assessed by qRT-PCR, and the ratio of mtDNA to nDNA is shown as a relative ratio. Flies were 1–2 days after eclosion. Data are expressed as mean ± SE, *n* = 3; *p* > 0.05; Student’s *t*-test. (**B**) Tau expression increases the activities of complexes I and IV. Mitochondria were extracted from 120 heads of flies with neuronal tau expression and subjected to Blue Native PAGE followed by in-gel activity assay to assess the activities of OXPHOS complexes I, II, IV, and V. Flies were 0–3 days after eclosion. Asterisk denotes supercomplex. (**C**) Tau expression upregulates the expression of genes encoding OXPHOS components. Extracts of heads with neuronal tau expression were subjected to qRT-PCR. Flies were 10 days after eclosion. Data are expressed as mean ± SE, *n* = 3; * *p* < 0.05, ** *p* < 0.01, *** *p* < 0.005, **** *p* < 0.0001; Student’s *t*-test. (**D**) Tau reduces mitochondrial distribution to the neurite. Electron microscopic analysis of mitochondria in the brain of the flies expressing tau in neurons at 50-day-old. The number of mitochondria (colored green) in the cell body region per cell (**left**) and those in the neuropil region per area (**right**) were analyzed. Representative images and quantitative analysis are shown. Data are expressed as mean ± SE, *n* = 3–12; * *p* < 0.05; one-way ANOVA followed by Dunnett’s test.

To determine whether the ATP reduction is caused by lower mitochondrial number, mtDNA levels were analyzed. Tau expression did not alter the relative mtDNA levels (Figure 3A), indicating that a reduction in ATP levels (Figure 2) is not due to loss of mitochondria.

A decrease in ATP levels and an increase in ROS levels may be due to defects in OXPHOS. We examined the activities of OXPHOS complexes in the heads of flies with or without neuronal expression of tau. Blue Native-PAGE (BN-PAGE) followed by in-gel activity assay (IGA) revealed that tau increases the activities of complexes I and IV (Figure 3B). We also noticed that tau increased supercomplexes containing complex I (Figure 3B, asterisk). The activities of complexes II and V were similar between the control and tau (Figure 3B). qRT-PCR revealed that neuronal expression of tau increases the expression of mRNA coding proteins in the OXPHOS complexes and cytochrome c (Figure 3C).

We also analyzed the morphology and distribution of mitochondria in the cell body region (Figure 3D, left) and neuropil region (Figure 3D, right) under a transmission electron microscope. The number of mitochondria in the neuropil region was lower in the tau-expressing fly brain (Figure 3D, right) but similar in the cell body region to that of the control (Figure 3D, left). Abnormality in mitochondrial morphology was not detected.

These results suggest that tau expression promotes the activity of OXPHOS complexes I and IV, which is consistent with higher ROS levels.

### 3.2. Effects of 5-ALA/SFC Feeding on Tau Toxicity

#### 3.2.1. 5-ALA/SFC Reduces Oxidative Damage Caused by Tau

We analyzed the effect of 5-ALA/SFC feeding on oxidative stress induced by tau. The brains of the flies expressing tau in neurons at 48 days after eclosion were stained with anti-4-HNE antibody to detect lipid peroxidation and ATP5A to detect mitochondria [40].

**Figure 4:**
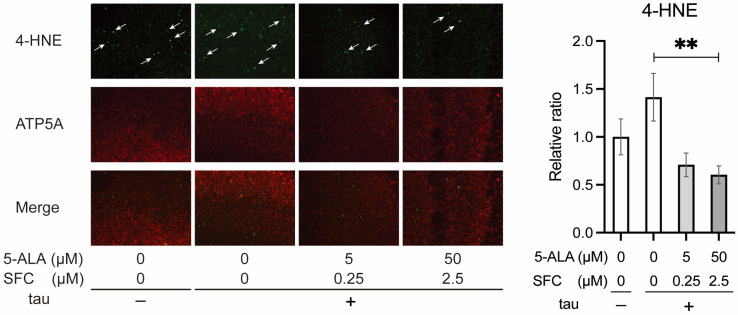
5-ALA/SFC reduces oxidative damage in the brains with tau expression. Immunostaining of the brains of driver-only control or flies with neuronal tau expression fed with 5-ALA and SFC at the indicated concentration with antibodies against a marker of lipid peroxidation, 4-HNE (green, arrows), and mitochondrial marker, ATP5A (red). Representative images and quantitative analysis are shown. Data are expressed as mean ± SE, *n* = 6–8; ** *p* < 0.01; one-way ANOVA followed by Dunnett’s test.

Tau expression tends to increase 4-HNE signals, although the difference did not reach statistical significance (*p* = 0.27, one-way ANOVA with Dunnett’s post hoc test). 5-ALA/SFC feeding reduced 4-HNE signals significantly (Figure 4). These results indicate that 5-ALA/SFC feeding lowers oxidative stress in tau-expressing neurons.

#### 3.2.2. 5-ALA/SFC Does Not Increase ATP Levels in Tau Flies

**Figure 5:**
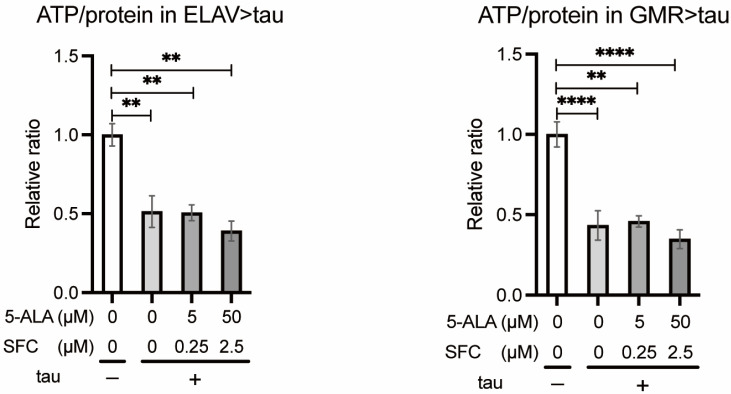
5-ALA/SFC does not increase ATP levels in tau flies. ATP levels in the heads of flies with tau expression in neurons at 46 to 49 days after eclosion (**left**) or those in the eye at 10 days after eclosion (**right**) were normalized with protein levels indicated as a relative ratio to the control. Data are expressed as mean ± SE, *n* = 3; ** *p* < 0.01, **** *p* < 0.0001; one-way ANOVA followed by Dunnett’s test.

We analyzed the effects of 5-ALA/SFC on ATP levels reduced by tau expression. ATP assays of head extracts of the flies expressing tau in neurons (ELAV > tau) or in the eyes (GMR > tau) revealed that 5-ALA/SFC feeding did not increase ATP levels (Figure 5).

#### 3.2.3. 5-ALA/SFC Does Not Correct Mitochondrial Distribution and OXPHOS Activities in Tau Flies

**Figure 6:**
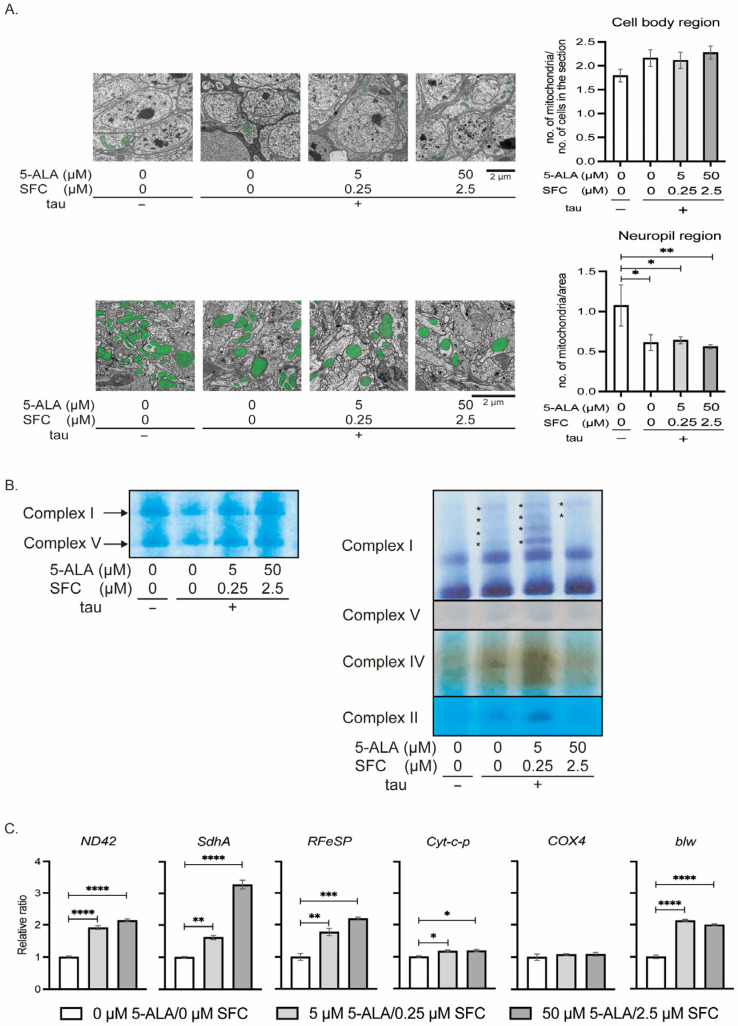
5-ALA/SFC does not correct mitochondrial distribution and OXPHOS activities in tau flies. (**A**) Ultrastructural analysis of heads of flies at 48 to 50 days after eclosion. The number of mitochondria (green) in the cell body region per cell (**top**) and that of the neuropil region per area (**bottom**) were analyzed. Representative images (**left**) and quantitative analysis (**right**). Data are expressed as mean ± SE, *n* = 3–12; * *p* < 0.05, ** *p* < 0.01; one-way ANOVA followed by Dunnett’s test. Data for flies without tau expression and those with tau expression without 5-ALA/SFC are the same as those shown in Figure 3D. (**B**) Mitochondria were extracted from 120 heads of tau flies and subjected to high-resolution Clear Native PAGE (**left**) and IGA (**right**). Asterisk (*) indicate supercomplexes with complex I activity. (**C**) Head extracts were subjected to qRT-PCR. Flies were at 10 days after eclosion. Data are expressed as mean ± SE, *n* = 3; * *p* < 0.05, ** *p* < 0.01, *** *p* < 0.005, **** *p* < 0.0001; one-way ANOVA followed by Dunnett’s test.

We analyzed the effects of 5-ALA/SFC on mitochondrial distribution and morphology in the brain. 5-ALA/SFC treatment did not affect the number of mitochondria in the cell body region (Figure 6A, top). Tau expression reduced mitochondrial number in the neuropil region, and 5-ALA/SFC treatment further reduced it in the neuropil region (Figure 6A, bottom).

We assessed the amount and activities of OXPHOS complexes by high-resolution Clear Native PAGE (hrCNE) and IGA. hrCNE revealed that the protein levels of complexes I and V were similar with or without 5-ALA/SFC feeding (Figure 6B, left). IGA showed that the elevated activities of complexes I, II, and IV in tau flies were further increased by 5-ALA/SFC feeding (Figure 6B, right). Also, expression of OXPHOS protein-coding genes in tau flies was further increased with 5-ALA/SFC feeding (Figure 6C).

#### 3.2.4. 5-ALA/SFC Reduces Tau Phosphorylation at Disease-Associated Sites and Mitigates Tau-Induced Neurodegeneration

**Figure 7:**
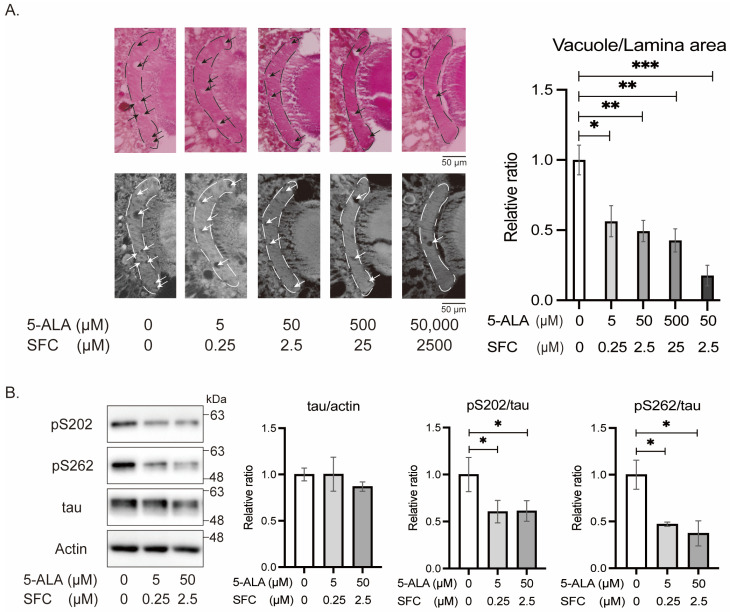
5-ALA/SFC reduces tau phosphorylation at disease-associated sites and mitigates tau-induced neurodegeneration. (**A**) Tau-induced neurodegeneration is observed as vacuoles in the lamina (arrows). (**Left**) Representative images (**top**: hematoxylin–eosin-stained sections; **bottom**: corresponding thresholded images used for quantification of vacuoles within the lamina, indicated by dashed lines). (**Right**) Quantification of vacuole area, normalized to the total lamina area. Flies were 10-day-old. Data are expressed as mean ± SE, *n* = 5; * *p* < 0.05, ** *p* < 0.01, *** *p* < 0.001; one-way ANOVA followed by Dunnett’s test. (**B**) Western blotting of head lysate with tau and phospho-tau specific antibodies. Actin was used as a loading control. Representative blots (**left**) and quantitative analysis (**right**). Data are expressed as mean ± SE, *n* = 3–4; * *p* < 0.05; one-way ANOVA followed by Dunnett’s test.

We analyzed the effects of 5-ALA/SFC on tau-induced neurodegeneration. Expression of tau in the retina causes degeneration of photoreceptor neurons, which is observed as vacuoles in the lamina, the first optic neuropil [28]. We found that 5-ALA/SFC feeding suppressed tau-induced neurodegeneration in a dose-dependent manner (Figure 7A).

We asked whether 5-ALA/SFC affects tau protein levels and tau phosphorylation at disease-associated sites. Western blotting of head lysates with a tau antibody and phospho-specific antibodies showed that 5-ALA/SFC feeding did not alter total tau levels, while phosphorylation at Ser202 and Ser262 was significantly reduced (Figure 7B).

## 4. Discussion

In this study, we examined the effects of tau on mitochondrial functions in neurons and tested the effects of 5-ALA/SFC using transgenic *Drosophila*. Six isoforms are expressed in the adult human brain (2N4R, 1N4R, 0N4R, 2N3R, 1N3R, 0N3R) [41], and all tau isoforms are found in aggregates in the AD brain [42]. We used a *Drosophila* model expressing 0N4R [25,43,44,45,46,47,48,49,50,51], in which tau-induced neurodegeneration is well characterized and widely used. We found that 5-ALA/SFC lowered oxidative damage, reduced tau phosphorylation, and mitigated neurodegeneration. Our results suggest that 5-ALA/SFC protects neurons against tau toxicity.

### 4.1. Tau Directly Modifies OXPHOS Complexes

We found that tau expression increases mito-roGFP2-Grx1 signal, indicating that mitochondrial ROS production is increased (Figure 1). In contrast, 4-HNE staining was not significantly different between control and tau-expressing flies, although a trend toward an increase was observed (Figure 4). This discrepancy likely reflects differences in the specificity and sensitivity of these detection methods. 4-HNE is a secondary product of lipid peroxidation and therefore represents an indirect measure of oxidative stress. Its accumulation depends not only on ROS levels but also on lipid composition, turnover, and detoxification pathways, which may limit its sensitivity to moderate ROS changes. In contrast, mito-roGFP2-Grx1 directly reports the glutathione redox state within mitochondria and is expressed specifically in neurons, providing a sensitive and compartment-specific readout. Thus, tau-induced ROS elevation may be sufficient to alter mitochondrial redox status without producing a statistically detectable increase in 4-HNE. Since mitochondrial ROS are primarily generated by complex I activity [7,10], elevated mitochondrial ROS are likely to be caused by enhanced complex I activity induced by tau expression (Figure 3). Tau-expressing flies exhibited decreased brain ATP (Figure 2), suggesting that enhanced activities of complexes I and IV and elevated OXPHOS gene expression in these fly brains (Figure 3) may be a compensatory response to reduced ATP.

Tau is known as a microtubule-associated protein, and its functions in mitochondria have been reported [52]. Comprehensive interactome analysis revealed direct interactions between tau and proteins located on the outer membrane, such as transporters; those on the inner membrane, including OXPHOS components and regulators; and enzymes in the matrix [53]. Tau interacts with complex I components such as NDUFS1 and NDUFS3; complex III components such as Cytochrome c1, SDHB, UQCRC1, and UQCRC2; complex IV components such as COX4I1, COX5A, COX5B, COX7A2; complex V components such as ATP5A1, ATP5B, ATP5C1, ATO5F1, ATP5H, ATP5J, ATP5J2, ATP5O, ATP6V1E1, ATP5EP2, and OXPHOS regulators C1QBP and ATPIF1; and tau with pathological mutations shows less interaction with some of these proteins [53]. Excess tau proteins may dysregulate the functions of these complexes. Tau proteins also interact with enzymes critical for the TCA cycle and amino acid metabolism, such as malate dehydrogenase, fumarate hydratase, glutamate dehydrogenase 1, and aspartate aminotransferase [53]. Thus, even with upregulation of OXPHOS complexes (Figure 3), dysregulation of these pathways can impair ATP synthesis. Tau interactions with mitochondrial antioxidant enzymes, such as mitochondrial peroxiredoxin, have been reported [53]. Although it is not clear how tau regulates these enzymes, excess tau may impair their functions and contribute to an increase in mitochondrial ROS.

### 4.2. 5-ALA/SFC Reduces Oxidative Damage

Elevated oxidative stress in tau flies was reduced by 5-ALA/SFC feeding (Figure 4). How does 5-ALA/SFC reduce oxidative damage? 5-ALA/SFC increased activities of complexes I, II, and IV (Figure 6B) and further increased OXPHOS transcripts in tau flies (Figure 6C). We also noticed that 5-ALA/SFC increased supercomplexes with complex I activity (Figure 6B). Supercomplexes have been proposed to contribute to efficient electron transfer with less ROS production [54,55,56]; thus, 5-ALA/SFC may mitigate oxidative damage via promoting supercomplex formation. 5-ALA/SFC has also been reported to bypass mitochondrial complex I deficiency by enhancing alternative electron entry to OXPHOS from complex II [23]. 5-ALA/SFC may shift metabolic programs to cope with the imbalance of OXPHOS and electron leak induced by tau.

### 4.3. 5-ALA/SFC Attenuates Tau Phosphorylation and Toxicity

**Figure 8:**
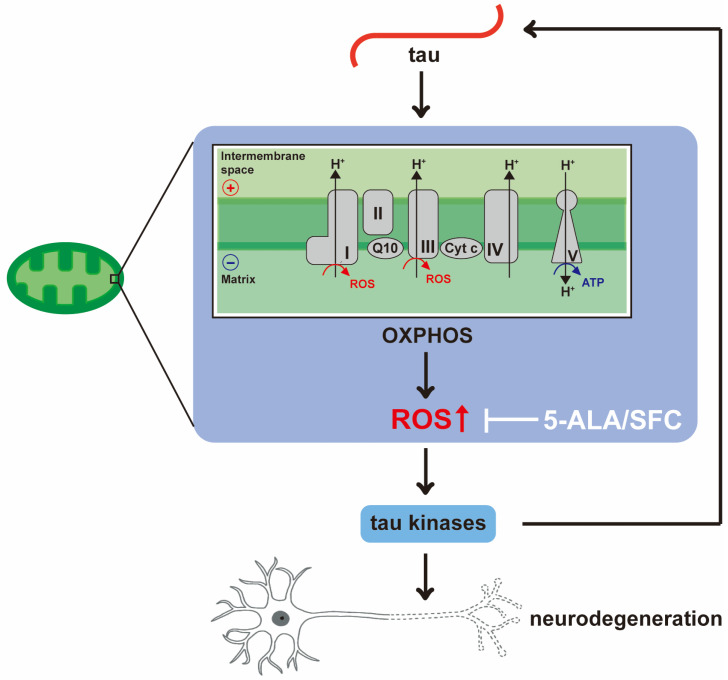
A model of a feed-forward mechanism of mitochondria, ROS and tau phosphorylation. Tau disrupts OXPHOS and increases mitochondrial ROS levels. Elevated ROS activates tau kinases, enhancing tau phosphorylation and further disrupting mitochondrial function. 5-ALA/SFC attenuates this feed-forward cascade by reducing ROS.

We found that 5-ALA/SFC suppresses tau-induced neurodegeneration (Figure 7A). ROS increase cellular vulnerability to tau [43,57]; thus, 5-ALA/SFC protects neurons by interacting with tau-induced cell death pathways. 5-ALA/SFC did not affect total tau levels, but it lowered tau phosphorylation at disease-associated sites (Figure 7B). Since tau phosphorylation at these sites, especially Ser262, is known to enhance tau-induced neurodegeneration [27,29,58,59], our results suggest that 5-ALA/SFC suppresses neurodegeneration via lowering neurotoxic tau species. ROS activate stress-induced kinases, such as MAPK, JNK, and GSK3β, which phosphorylate tau at Ser and Thr followed by proline, including Ser202 [60]. Ser262, in a KXGS motif in a microtubule-binding repeat, is not followed by proline [61] and is not phosphorylated by these proline-directed kinases [61]. Instead, Ser262 is phosphorylated by AMPK family members [62,63,64,65]. Mitochondrial ROS have been reported to activate AMPK [66], and 5-ALA/SFC may lower AMPK activity via reduction of ROS in tau flies. Ser262 is also phosphorylated by checkpoint kinase 2 (Chk2), which is activated by DNA damage [67]. ROS-induced DNA damage, and the following activation of Chk2, may be lowered by 5-ALA/SFC. Our results suggest that tau, mitochondria, and ROS form a feed-forward mechanism leading to neurodegeneration, and 5-ALA/SFC mitigates this mechanism via decreasing ROS (Figure 8).

## 5. Conclusions

Our study revealed the functional interaction between tau and the mitochondrial OXPHOS complex and the protective effects of 5-ALA/SFC in vivo. Our results suggest that 5-ALA/SFC protects against tau toxicity via lowering ROS and tau phosphorylation. Further investigation into these pathways will advance our understanding of tau toxicity and the mechanisms of the neuroprotective action of 5-ALA/SFC.

## Figures and Tables

**Table 1:** Fly genotypes used in this study.

Figure 1	control	*elav-Gal4/Y;UAS-mito-roGFP2-Grx1^9^/+;UAS-luciferase/+*
	tau	*elav-Gal4/Y;UAS-mito-roGFP2-Grx1^9^/+;UAS-tau/+*
Figure 2	control	*elav-Gal4/Y;UAS-tdTomato/+;+/+*
	tau	*elav-Gal4/Y;+/+;UAS-tau/+*
Figure 3A	control	*elav-Gal4/Y;+/+;+/+*
	tau	*elav-Gal4/Y;+/+;UAS-tau/+*
Figure 3B	control	*elav-Gal4/Y;+/+;+/+*
	tau	*elav-Gal4/Y;UAS-tau/CyO;+/+*
Figure 3C	control	*elav-Gal4/Y;+/+;+/+*
	tau	*elav-Gal4/Y;+/+;UAS-tau/+*
Figure 3D	control	*+/+;gmr-Gal4/+;+/+*
	tau	*+/+;gmr-Gal4/+;UAS-tau/+*
Figure 4	tau −	*elav-Gal4/Y;+/+;+/+*
	tau +	*elav-Gal4/Y;+/+;UAS-tau/+*
Figure 5	tau −	*elav-Gal4/Y;+/+;+/+* *+/Y;gmr-Gal4/+;+/+*
	tau +	*elav-Gal4/Y;+/+;UAS-tau/+* *+/Y;gmr-Gal4/+;UAS-tau/+*
Figure 6A	tau −	*+/+;gmr-Gal4/+;+/+*
	tau +	*+/+;gmr-Gal4/+;UAS-tau/+*
Figure 6B	tau −	*+/+;gmr-Gal4/+;+/+*
	tau +	*+/+;gmr-Gal4/+;UAS-tau/+*
Figure 6C		*+/+;gmr-Gal4/+;UAS-tau/+*
Figure 7		*+/+;gmr-Gal4/+;UAS-tau/+*
Figure 8		*+/+;gmr-Gal4/+;UAS-tau/+*

## Data Availability

The original contributions presented in this study are included in the article. Further inquiries can be directed to the corresponding author.
